# Asymptomatic people with well-controlled HIV do not have abnormal left ventricular global longitudinal strain

**DOI:** 10.3389/fcvm.2023.1198387

**Published:** 2023-07-20

**Authors:** Jennifer F. Hoy, Sue J. Lee, Janine M. Trevillyan, Elizabeth M. Dewar, Janine Roney, Anthony Dart, Yan Yang

**Affiliations:** ^1^ Department of Infectious Diseases, Alfred Health and Monash University, Melbourne, VIC, Australia; ^2^ Department of Cardiology, Alfred Health, Melbourne, VIC, Australia; ^3^ Alfred Baker Medical Unit, Baker Heart and Diabetes Institute, Melbourne, VIC, Australia

**Keywords:** HIV, cardiovascular disease, global longitudinal strain, echocardiogram, left ventricular systolic function

## Abstract

**Background:**

Previous studies have reported impairment in systolic and diastolic function in people with HIV (PWHIV). Our aim was to determine if echocardiographically measured left ventricular (LV) global longitudinal strain (GLS) is abnormal in asymptomatic PWHIV.

**Methods:**

A cross-sectional study of PWHIV (*n* = 98, 89% male, median age 53 years) and HIV-negative people (*n* = 50, median age 53 years) without known cardiovascular disease were recruited from a single centre. All participants completed a health/lifestyle questionnaire, provided a fasting blood sample, and underwent a comprehensive echocardiogram for assessment of diastolic and systolic LV function, including measurement of GLS.

**Results:**

All PWHIV were receiving antiretroviral therapy (ART) for a median of 12 years (IQR: 6.9, 22.4), the majority with good virological control (87% suppressed) and without immunological compromise (median CD4 598 cells/µl, IQR: 388, 841). Compared with controls of similar age and gender, there was no difference in GLS [mean GLS −20.3% (SD 2.5%) vs. −21.0% (SD 2.5%), *p* = 0.14] or left ventricular ejection fractions [65.3% (SD 6.3) vs. 64.8% (SD 4.8), *p* = 0.62]. Following adjustment for covariates (gender, heart rate, systolic and diastolic blood pressure, and fasting glucose), the difference in GLS remained non-significant. There were no differences in LV diastolic function between the groups. Exposure to at least one mitochondrially toxic ART drug (didanosine, stavudine, zidovudine, or zalcitabine) was not associated with impairment of LV systolic function.

**Conclusion:**

No clinically significant impairment of myocardial systolic function, as measured by LV GLS, was detected in this predominantly Caucasian male population of PWHIV on long-term ART, with no history of cardiovascular disease.

## Introduction

The success of antiretroviral therapy (ART) has shifted the course of HIV to one of near-normal life expectancy, albeit with an increased prevalence of serious non-AIDS conditions, including cardiovascular disease. People with HIV (PWHIV) are at increased risk for atherosclerotic cardiac disease (1, 2), and clinical heart failure (3, 4), with some reports of an association with specific antiretroviral therapy drugs (5, 6). A number of, although not all, echocardiographic studies in PWHIV have shown an increased prevalence of left ventricular (LV) systolic and/or diastolic dysfunction (7–11). Recent cardiac magnetic resonance imaging (CMR) studies have also shown left ventricular systolic and/or diastolic dysfunction in some (12–14) but not all studies (15, 16). A large study evaluating cardiac chamber volumes in almost 600 PWHIV who were virologically suppressed on ART, and age-and sex-matched controls, using multidetector CT scans to measure cardiac volumes which were then indexed to body surface area (BSA) was reported. In this study, HIV was independently associated with minor reductions in LV diastolic volume. The minor differences in RV diastolic volume and LV mass were no longer significant after controlling for BSA (17). Pulmonary hypertension is also well recognised as a complication of poorly controlled HIV and ART use (18, 19). The aetiology of heart failure associated with HIV is not fully elucidated (20), and although it is more common after acute myocardial infarction in PWHIV (21), the increased incidence of heart failure in PWHIV is not explained solely by the increased risk of coronary disease. A number of other mechanisms, including myocarditis and fibrosis, have been implicated (22, 23). Whilst CMR may be more informative than echocardiography in defining cardiac pathophysiology, it is not feasible to be used as a screening tool to be applied to ambulatory people with HIV. As noted by Arendt et al., “Transthoracic echocardiography (TTE) is the primary tool for assessing myocardial function. It is cost effective, readily available, and robust in identifying both systolic and diastolic function” (24).

The majority of echocardiographic studies examining left ventricular systolic function in PWHIV have been limited to evaluation of changes in left ventricular ejection fraction and ventricular volumes. The use of left ventricular global longitudinal strain (GLS) offers the possibility of identifying a subgroup of PWHIV who have not yet manifested clinical evidence of heart disease but are developing cardiac dysfunction not evident from measurement of LV ejection fraction. Identification of such individuals could allow early-targeted intervention to prevent further progression. The earlier studies in small patient groups included measurement of GLS in PWHIV (25, 26). More recently, a larger study in a young population (mean age 14 years) reported abnormal GLS in comparison with population norms and related this to virological suppression and use of zidovudine (27). A retrospective study in adults with HIV referred for echocardiography also examined the relationship between GLS and immune status, with greater immunodeficiency inversely correlated with GLS (28). Finally, a study in asymptomatic US soldiers with very recent acquisition of HIV, with high HIV viral loads, and not on antiretroviral treatment found no evidence of LV systolic dysfunction or reduced GLS (29).

The primary objective of this study was to determine the potential value of screening for subclinical cardiac dysfunction in adults with HIV. We assessed cardiac function, as estimated primarily by GLS, detected in adults with well-controlled HIV on antiretroviral treatment compared with an HIV-negative control group of similar age, ethnicity, and sex. In addition, we examined the factors, including ART, that may contribute to cardiac dysfunction in PWHIV.

## Methods

### Study population

This single centre study recruited PWHIV, aged between 30 and 70 years, when they attended the Infectious Diseases outpatient clinic for routine HIV care. A convenience sample of HIV-negative healthy volunteers of similar age (+/− 5 years) and sex was recruited *via* advertising. All participants with HIV were receiving ART. Individuals were excluded if they had a history of myocardial infarction, cardiac surgery, atrial fibrillation, significant arrhythmia or known valvular heart disease, in addition to newly diagnosed valvular heart disease detected during screening.

The participants attended for a single research visit following an overnight fast. At this visit, transthoracic echocardiography was performed, medical history was taken (and confirmed with existing medical records), blood pressure was measured with Philips Suresigns VS3 after a 5 min rest, and blood was collected for markers of HIV control (HIV viral load, CD4 cell count), renal function, glucose levels, and lipids [total cholesterol, high-density lipoprotein cholesterol (HDLc), low-density lipoprotein cholesterol (LDLc), and triglycerides]. Hypertension was defined as a systolic blood pressure above 140 mmHg, diastolic blood pressure above 90 mmHg, or prescription of antihypertensive medication. The duration of known HIV infection and the duration of ART were determined as the time from first positive HIV serology or time of ART start to study visit, respectively. An undetectable HIV viral load was defined as <20 copies/ml.

The participants completed a questionnaire concerning lifestyle factors such as smoking, drug use, diet, alcohol consumption, and usual exercise levels, as well as symptoms potentially related to heart failure.

### Echocardiogram protocol

Comprehensive transthoracic echocardiography was performed using a cardiac ultrasound system (iE33 ultrasound system, Royal Dutch Philips Electronics, Bothell, WA, USA) by a single echocardiography-trained cardiologist. Two-dimensional data acquisitions were obtained from parasternal long-axis and short-axis views and the three standard apical views. For each view, three consecutive cardiac cycles were recorded. The LV diameters and the interventricular septal wall and posterior wall thickness were measured at end-diastole from M-mode recordings according to the guidelines of the American Society of Echocardiography (30, 31). LV mass in grams was calculated from M-mode echocardiograms according to the formula described by Devereux et al. (32). LV mass was indexed to body surface area as LV mass index (LVMI) in g m^−2^ BSA. Biplane disc summation method was used to calculate left atrial (LA) volumes in the four-chamber views, and these values were indexed by BSA as LA volume index (LAVI) in g m^−2^ BSA. The LV volumes were measured by biplane 2D Simpson's method, and LV ejection fraction was calculated using standard equations.

The mitral valve inflow peak early filling velocity (MVE) and deceleration time (MVDT) of the early filling velocity were assessed by pulsed-wave Doppler in the apical four-chamber view. Tissue Doppler Imaging (TDI) was also performed in the apical four-chamber view. The early diastolic annular velocity (e') was measured by TDI recordings. For the assessment of global LV diastolic function, Tissue Doppler signals at the septal and lateral sides of the mitral annulus were acquired, and their mean values were calculated, providing early diastolic Tissue Doppler mean velocity (e' mean). The ratio of MVE to e' mean (MV E/e') was then calculated. Diastolic dysfunction was defined if 3 or more of e’, MV E/e’ ratio, LAVI, and peak tricuspid regurgitation (TR) velocity were abnormal.

All data of speckle tracking were analysed offline using a dedicated automated software (Qlab). Three endocardial markers were placed in an end diastolic frame at apical 4-, 2-, and 3-chamber views for the 2D longitudinal speckle tracking analysis. The software automatically tracked the contour of endocardium to cover the myocardial thickness of the entire LV wall. Appropriate tracking was corroborated in real time and manually adjusted for the area of interest to ensure accurate tracking. The mean LV global GLS was calculated for the 18 segments (6 basal, 6 mid, and 6 apical) in relation to the strain magnitude at aortic valve closure. A single observer made LV GLS measurements. The coefficient of variation (CV) for repeated measures was <2%.

### Statistical analysis

For the sample size estimation, the published average GLS of −19.7% (CI −20.4 to −18.9) in healthy people without known cardiovascular disease (33) or HIV was used with a standard deviation of 5, which was approximately twice that found in males from a European single centre study (34). The standard deviation was doubled to account for the fact that our population was older and our sample size smaller. Assuming that PWHIV would have a proportional increase of 15% in GLS and using a ratio of 2:1 with alpha 0.05, 100 PWHIV:50 HIV-negative people provided 90% power to detect a difference between groups.

The participant demographics, echocardiography variables, and lifestyle factors were described using frequencies (%), mean (standard deviation, SD), and median (interquartile range, IQR), depending on distribution. Unadjusted comparisons between individuals with and without HIV were made using a *χ*^2^ or Fisher's exact test, Student's *t*-test, or Mann–Whitney *U* test, as appropriate.

The primary outcome was the mean difference (SD) in LV GLS between individuals with and without HIV. Secondary outcome measures included differences in echocardiographic measures of LV systolic and diastolic function and right heart function.

To determine the factors that might impact the relationship between LV GLS and HIV, the participant demographic, lifestyle variables, and echocardiographic variables with a *p*-value less than 0.05 from the unadjusted analysis were included in a multivariable model, with the *a priori* specified variables of gender and systolic and diastolic blood pressure. Using a stepwise selection procedure, only variables that were significant at *p* < 0.05 were retained in the final model.

All analyses were performed using STATA, v17.0 (College Station, TX, USA).

All participants provided written informed consent. The study was approved by the Alfred Hospital Ethics Committee (Project: #240/17).

## Results

PWHIV (*n* = 100) and HIV-negative controls (*n* = 50) were recruited. Two PWHIV were subsequently excluded because they were found to have significant valvular disease on echocardiography; thus, 98 PWHIV were analysed. The participants with HIV were predominantly male [90 (91.8%)] with a median age of 53 years and Caucasian [82 (83.7%)], reflecting the demographic of PWHIV receiving HIV care at this centre.

PWHIV were more likely to be current or ex-smokers and were more likely to be on antihypertensive or lipid lowering medications and had lower HDLc levels, higher triglyceride levels, and higher resting heart rates than HIV-negative controls (Table 1). PWHIV were lighter, with lower body mass index (BMI) and were more likely to have evidence of previous or current infection with hepatitis C (Table 1).

**Table 1:** Participant demographics.

	Overall	HIV negative	PWHIV	*p*-Value*
*N*	**148**	**50**	**98**	
Age, years	53.0 (45.0, 59.5)	53.0 (42.0, 62.0)	53.0 (47.0, 59.0)	0.79
Male	132 (89.2%)	42 (84.0%)	90 (91.8%)	0.15
Caucasian race	127 (85.8%)	45 (90.0%)	82 (83.7%)	0.17
Hypertension^a^	28 (18.9%)	2 (4.0%)	26 (26.5%)	**<0** **.** **001**
Diabetes	6 (4.1%)	0 (0.0%)	6 (6.1%)	0.074
Smoking status				**<0** **.** **001**
Never	79 (53.4%)	38 (76.0%)	41 (41.8%)	** **
Past	42 (28.4%)	11 (22.0%)	31 (31.6%)	
Current	26 (17.6%)	1 (2.0%)	25 (25.5%)	
Missing	1 (0.7%)	0 (0.0%)	1 (1.0%)	
Family history of CVD^b^	67 (45.3%)	23 (46.0%)	44 (44.9%)	0.90
Heart rate, BPM	60 (55, 65)	55.5 (53, 60)	62 (56, 70)	**<0** **.** **001**
Systolic BP, mmHg	129.7 (14.8)	129.6 (14.3)	129.8 (15.2)	0.95
Diastolic BP, mmHg	82.4 (9.3)	84.0 (8.4)	81.6 (9.7)	0.14
BMI, kg/m^2^	26.6 (3.9)	28.1 (3.6)	25.8 (3.9)	**<0** **.** **001**
Renal dysfunction (eGFR ≤ 60 ml/min)	7 (4.9%)	0 (0.0%) *n* = 44	7 (7.1%)	0.069
Lipids, mmol/L				
Total cholesterol	5.4 (4.7, 6.1)	5.75 (4.8, 6.35) *n* = 48	5.3 (4.7, 6.1)	0.19
HDLc	1.3 (1.1, 1.5)	1.4 (1.2, 1.8) *n* = 49	1.2 (1, 1.4)	**<0** **.** **001**
LDLc	3.4 (2.7, 4.1)	3.65 (2.65, 4.35) *n* = 48	3.3 (2.7, 3.8) *n* = 94	0.22
Triglycerides	1.3 (0.9, 2)	1.1 (0.8, 1.6) *n* = 48	1.4 (0.9, 2.2)	**0** **.** **016**
Fasting glucose (mmol/L)	4.8 (4.5, 5.2)	4.6 (4.1, 5) *n* = 45	4.9 (4.6, 5.4) *n* = 95	**<0** **.** **001**
Hepatitis C^c^	9 (6.2%)	0 (0.0%) *n* = 48	9 (9.2%)	**0** **.** **030**
Concomitant medications				
Antihypertensive	26 (17.6%)	4 (8.0%)	22 (22.4%)	**0** **.** **029**
Antiplatelet	2 (1.4%)	0 (0.0%)	2 (2.0%)	0.31
Lipid lowering	36 (24.3%)	7 (14.0%)	29 (29.6%)	**0** **.** **037**
HIV-specific variables				
Undetectable HIV viral load^d^			85 (87%)	
CD4 cell count, cells/µl			598.5 (388, 841)	
Nadir CD4 cell count, cells/µl			168 (63, 320)	
Duration of HIV, years			13.9 (8.5, 24.0)	
Duration of ART, years			11.9 (6.9, 22.4)	
Current ART				
NNRTI			33 (34%)	
Integrase inhibitor			63 (64%)	
Protease inhibitor			23 (23%)	
Tenofovir^e^			60 (61.2%)	
Abacavir			18 (18%)	

Data are presented as mean (SD) or median (p25, p75) for continuous measures, depending on distribution, and *n* (%) for categorical measures. Bold values denote the statistically significant.

ART, antiretroviral therapy; BMI, body mass index; BPM, beats per minute; BP, blood pressure; CVD, cardiovascular disease; eGFR, estimated glomerular filtration rate; HDLc, high-density lipoprotein cholesterol; LDLc, low-density lipoprotein cholesterol; NNRTI, non-nucleoside reverse transcriptase inhibitor; PWHIV, people with HIV.

* *p*-values calculated by Wilcoxon rank-sum or Student's *t*-test for continuous variables (as appropriate) and Pearson’s *χ*^2^ test for categorical variables.

^a^ Defined as a systolic BP > 140 mmHg or diastolic BP > 90 mmHg, or taking antihypertensive medication.

^b^ In a first degree relative <55 years of age at the time of diagnosis.

^c^ Determined by hepatitis C antibody positivity.

^d^ Defined as <20 copies/ml.

^e^ Combination of tenofovir disoproxil fumarate (*n* = 19) and tenofovir alafenamide (*n* = 41).

PWHIV had a long median duration of HIV infection [13.9 years (IQR: 8.5–24 years)], were all on ART, largely with a suppressed viral load (87%), and had good immunological function [median CD4 cell count, 598 cells/µl (IQR: 388–841)] (Table 1). Those with detectable HIV viral loads (*n* = 13) had low level viremia with a median HIV RNA of 35 (IQR: 27–35) copies/ml.

### Echocardiography findings

In univariate analyses, PWHIV had higher mean heart rates (62 beats per minute vs. 55.5 beats per minute *p* < 0.001, Table 1). There was no difference in mean systolic or diastolic blood pressures between groups. The mean (SD) LV GLS was −20.3% (2.5) in PWHIV and −21.0% (2.5) in people without HIV (Table 2). The mean LV GLS was not different between groups [mean difference 0.65 (95% CI: 0.21–1.50), *p* = 0.137]). One patient in the HIV group had LV GLS higher than −15 (LV GLS = −13.5), giving a prevalence of subclinical cardiac dysfunction, as estimated by GLS, of 0.68%.

**Table 2:** Echocardiography findings in the participants with and without HIV.

	Overall	HIV negative	PWHIV	*p*-Value*
*n*	148	50	98	
Left ventricular systolic function measures				
LV global longitudinal strain, %	−20.5 (2.5)	−21.0 (2.5)	−20.3 (2.5)	0.14
LV ejection fraction, %	65.2 (5.8)	64.8 (4.8)	65.3 (6.3)	0.62
Left ventricular diastolic function measures				
MV E/e’ mean	8.7 (2.3) *n* = 146	8.4 (2.1) *n* = 49	8.9 (2.3) *n* = 49	0.16
MV deceleration time, ms	0.2 (0.0)	0.2 (0.0)	0.2 (0.1)	0.78
Left heart structure and volumes				
LV mass index, g/m^2^	79.6 (17.7)	86.3 (15.8)	76.1 (17.8)	**<0.001**
LV end systolic volume, ml	39.0 (12.7)	37.3 (10.4)	39.8 (13.7)	0.27
LV end diastolic volume, ml	110.2 (25.4)	105.8 (25.8)	112.5 (25.0)	0.13
Left atrial volume index, ml/m^2^	28.7 (8.5)	34.3 (8.0)	25.8 (7.3)	**<0.001**
Pulmonary artery pressure				
PAP, mmHg	18.8 (6.7) *n* = 131	18.7 (6.7) *n* = 36	18.9 (6.7) *n* = 95	0.88

LV, left ventricle; MV, mitral valve; MV E/e’, ratio of mitral valve E wave velocity/mean e’ velocity; PAP, pulmonary artery systolic pressure.

Data are presented as mean (SD). Bold values denote the statistically significant.

* *p*-Values are calculated by the Student's *t*-test.

There was no difference in the LV ejection fraction between groups [mean difference 0.51 (95% CI: 1.50–2.52), *p* = 0.62], and LV ejection fraction was inversely correlated with GLS (Pearson's correlation coefficient = −1.18, *p* = 0.03). PWHIV demonstrated less evidence of early systolic dysfunction with lower LV mass [76.1 g/m^2^ (SD: 17.8) vs. 86.3 g/m^2^ (SD: 15.8), *p* < 0.001, Table 2] and significantly lower left atrial volume indexed for body surface area (LAVI), *p* < 0.001. Mitral valve deceleration time and mean MV E/e' were similar between groups. There were also no differences between groups in the transmitral valve flow profiles E or A wave amplitudes, nor in e' septal, e' lateral, e' mean velocities, MV E/e' septal, or MV E/e' lateral (*p* > 0.1 for all, data not shown).

Demographic and self-reported lifestyle variables that were significantly associated with HIV were included in a multivariable logistic regression model with LV GLS as the main covariate of interest. The initial model included recreational drug use in the past 6 months, vigorous physical activity for at least 10 min at a time in the last 7 days, current smokers, heart rate, BMI, HDL cholesterol level, triglyceride level, fasting glucose levels, use of antihypertensive medication, and use of lipid lowering medication. The activities limited by breathlessness and hepatitis C were not included in the model as all cases for both of these factors occurred in the PWHIV group. The echocardiographic variables LVMI and LAVI were also considered in the model, and the model was adjusted for gender and systolic and diastolic blood pressure. The adjusted odds (95% CI) of LV GLS in PWHIV was 1.08 (0.80, 1.45), *p* = 0.628.

In PWHIV, there was a borderline significant correlation between GLS and log_10_ CD4 cell count [coefficient 0.79 (95% CI: 0.12, 1.45) *p* = 0.022]; however, this was no longer significant after adjustment for covariates (smoking, recreational drug use, fasting glucose, cholesterol, HDL, BMI, heart rate, systolic BP, diastolic BP, sex, and left ventricular mass index). There was no association between log_10_ CD8 cell count nor viral load with GLS in PWHIV.

There was significantly higher recreational drug use in PWHIV (*p* = 0.027). PWHIV were less likely to have undertaken vigorous activity in the last week (*p* = 0.022) and were more likely to report shortness of breath that was limiting their usual activity (*p* = 0.019). There was no difference in the reported incidence of peripheral oedema, positional dyspnoea, or difficulty climbing stairs (Table 3).

**Table 3:** Lifestyle factors and reported symptoms in people with and without HIV.

	Overall	HIV negative	PWHIV	*p*-Value*
*N*	**148**	**50**	**98**	** **
Number of standard alcohol drinks per day, *n* (%)				0.057
None	14 (9.5%)	0 (0.0%)	14 (14.3%)	
1–2 drinks	81 (54.7%)	31 (62.0%)	50 (51.0%)	
3–6 drinks	46 (31.1%)	17 (34.0%)	29 (29.6%)	
7+ drinks	5 (3.4%)	2 (4.0%)	3 (3.1%)	
*Missing*	2 (1.4%)	0 (0.0%)	2 (2.0%)	
Recreational drug use				
Use of any recreational drugs in the past 6 months? (includes illicit use of legal drugs)	26 (17.7%) *n* = 147	4 (8.0%)	22 (22.7%) *n* = 97	0.27
Ever injected drugs	5 (3.4%) *n* = 147	0 (0.0%)	5 (5.2%) *n* = 97	0.10
Physical activity during the last 7 days				
Any VIGOROUS physical activity for at least 10 min at a time	68 (46.9%) *n* = 145	30 (60.0%)	38 (40.0%) *n* = 95	**0**.**022**
Any MODERATE physical activity for at least 10 min at a time	101 (68.6%) *n* = 147	34 (68.0%)	67 (69.1%) *n* = 97	0.89
Any WALKING physical activity for at least 10 min at a time	141 (95.9%) *n* = 147	48 (96%)	93 (95.9%) *n* = 97	0.97
Reported symptoms				
Are your activities limited by breathlessness?	10 (6.8%) *n* = 147	0 (0.0%)	10 (10.3%) *n* = 97	**0.019**
Do your feet or ankles become swollen by the end of the day?	10 (6.8%) *n* = 147	1 (2.0%)	7 (7.2%) *n* = 97	0.097
Do you get breathless when undertaking normal daily activities?	8 (5.4%) *n* = 147	1 (2.0%)	7 (7.2%) *n* = 97	0.19
Can you climb two flights of stairs without getting breathless?	119 (81.0%) *n* = 147	42 (84.0%)	77 (79.4%) *n* = 97	0.50
Do you get breathless when sitting in bed?	4 (2.7%) *n* = 146	0 (0.0%)	4 (4.2%) *n* = 96	0.14
Do you get breathless when lying in bed?	4 (2.7%) *n* = 147	0 (0.0%)	4 (4.2%) *n* = 97	0.15

PWHIV, people with HIV.

Data are presented as *n* (%) or median (IQR). Bold values denote the statistically significant.

* *p*-Values are calculated by *χ*^2^ or Wilcoxon rank-sum test.

### Impact of older antiretroviral therapy on echocardiogram findings

A total of 49 (50%) people had been exposed to older, mitochondrially toxic antiretrovirals (any of didanosine, zalcitabine, zidovudine, or stavudine) (Table 4). PWHIV who had been exposed to these older agents were older (57 years vs. 47 years, *p* < 0.001), had a longer duration of ART (22.4 years vs. 6.9 years, *p* < 0.001), and a median of 10.0 years (IQR: 5.6, 13.9) exposure to the older ART drugs. They had higher mean systolic (136 mmHg vs. 123 mmHg) and diastolic (85 mmHg vs. 78 mmHg) blood pressures, and a lower mean BMI [26.6 (SD: 4.1) kg/m^2^ vs. 25.0 (SD: 3.6) kg/m^2^, *p*-value = 0.044] than those who had not (Table 4). They were also less likely to be current smokers [9 (18.4%) vs. 16 (32.7%), *p* = 0.016].

**Table 4:** Demographic and echocardiography findings in the participants with HIV (*n* = 98), by exposure to older mitochondrial toxic ART.

	Overall	Contemporary ART	Old ART^a^ exposure	*p*-Value*
*n*	98	49	49	
Age, years	53.0 (47.0, 59.0)	47.0 (41.0, 61.0)	57.0, 53.0, 61.0)	**<0** **.** **001**
BMI, kg/m^2^	25.8 (3.9)	26.6 (4.1)	25.0 (3.6)	**0**.**044**
Heart rate, beats/min	62 (56, 70)	62 (60, 70)	60 (55, 66)	0.14
Systolic BP, mmHg	129.8 (15.2)	123.5 (10.8)	136.0 (16.4)	**<0**.**001**
Diastolic BP, mmHg	81.6 (9.7)	78.2 (9.0)	85.0 (9.3)	**<0**.**001**
Smoking status				**0**.**016**
Never	42 (43%)	24 (49%)	18 (37%)	
Past	31 (32%)	9 (18%)	22 (45%)	
Current	25 (26%	16 (33%)	9 (18%)	
HIV-specific variables				
Undetectable HIV VL	85 (87%)	43 (88%)	42 (86%)	0.77
CD4 cell count (cells/µl)	598.4 (388, 841)	627 (516, 862)	527 (354, 722)	0.052
Nadir CD4 cell count (cells/µl)	168 (63, 320)	271 (58, 413)	137 (63, 189)	**0**.**006**
Duration of HIV, years	13.9 (8.5, 24.0)	8.7 (4.9, 11.3)	24.0 (18.1, 31.1)	**<0**.**001**
Duration of ART, years	11.9 (6.9, 22.4)	6.9 (4.6, 9.9)	22.4 (16.8, 25.6)	**<0**.**001**
Left ventricular systolic function measures				
LV global longitudinal strain, %	−20.3 (2.5)	−19.9 (2.6)	−20.7 (2.3)	0.090
LV ejection fraction, %	65.3 (6.3)	63.9 (5.9)	66.8 (6.4)	**0**.**023**
Left ventricular diastolic function measures				
MV E/e’ mean	8.9 (2.3) *n* = 97	8.3 (2.3) *n* = 48	9.5 (2.2)	**0**.**007**
MV deceleration time, ms	0.2 (0.1)	0.2 (0.1)	0.2 (0)	0.14
Left heart structure and volumes				
LV mass index, g/m^2^	76.1 (17.8)	71.9 (15.0)	80.3 (19.4)	**0**.**018**
LV end systolic volume, ml	39.8 (13.7)	42.5 (14.0)	37.1 (12.9)	**0**.**049**
LV end diastolic volume, ml	112.5 (25.0)	115.5 (25.1)	109.5 (24.9)	0.24
Left atrial volume index, ml/min^2^	25.8 (7.3)	25.1 (7.0)	26.5 (7.6)	0.32
Pulmonary artery pressure				
PAP, mmHg	18.9 (6.7) *n* = 95	17.8 (5.5) *n* = 47	20.0 (7.6) *n* = 48	0.11

Data are presented as mean (SD) or median (p25, p75) for continuous variables, depending on distribution, and *n* (%) for categorical variables. Bold values denote the statistically significant.

ART, antiretroviral therapy; BMI, body mass index; LV, left ventricular; MV E/e’, ratio of mitral valve E wave velocity/mean e’ velocity; MV, mitral valve; PAP, pulmonary artery systolic pressure.

* *p*-values calculated by Wilcoxon rank-sum test or Student's *t*-test for continuous variables (as appropriate) and using the *χ*^2^ test for categorical variables.

^a^ Defined as ever prescribed zidovudine, zalcitabine, didanosine, or stavudine.

When comparing PWHIV who had or had not been exposed to older mitochondrial toxic antiretroviral therapy (any of stavudine, zidovudine, didanosine, or zalcitabine), we found no difference in mean GLS (Table 4). Those exposed to older ART agents had a higher LVEF; however, LVEF for both groups was within the normal range. They also had higher ratio of mitral valve E velocity/e’ velocity (MV E/e’), which correlates with left atrial pressure; however, these measures were also within the normal range. Systolic and diastolic blood pressures and LV mass were significantly higher, and LV end systolic volume was significantly lower in the PWHIV group.

Because there was a significant age difference between those PWHIV who had or had not been exposed to the older ART, multivariate regression was performed adjusting for age to determine if this was driving the differences in the echocardiography parameters detected. After adjustment, the association between GLS and PWHIV exposed to older ART was significant indicating that those exposed to older ART had significantly lower GLS [OR (95% CI) of 0.79 (0.65, 0.96), *p* = 0.018]. A similar association was found for LVEF [OR (95% CI) of 1.09 (1.01, 1.17), *p* = 0.033]. There was no alteration to the directionality of the results. When adjustment for the other factors significant on univariate analysis were included, the association between GLS and exposure to older ART became non-significant, whilst the association between LVEF and exposure to older ART remained borderline significant [OR (95% CI) 1.1 (1.0, 1.19), *p* = 0.04].

## Discussion

We found no difference in mean GLS values between people with well-controlled HIV infection and controls, with all GLS values measured, except one in a person with HIV, within the normal range in adults (33). PWHIV had a significantly higher heart rate, but there was no difference in systolic or diastolic blood pressure, diastolic function, or left ventricular ejection fraction between groups. However, PWHIV had significantly higher rates of hypertension (defined as systolic BP > 140 mmHg, diastolic BP > 90 mmHg, or use of antihypertensive medication). This is in contrast to some other studies who reported a higher prevalence of abnormal GLS, and cardiac dysfunction (11, 28, 35), but these studies were retrospective analyses of echocardiograms performed in people referred for assessment, suggesting a biased potentially symptomatic population. The studies in prospectively recruited cohorts with or without HIV-negative control groups were less likely to report clinically significant changes in GLS and other parameters of cardiac function (17, 29).

In PWHIV in our study, there was also no difference in GLS between those who had received “older” mitochondrially toxic ART compared with those who had not, whilst left ventricular ejection fraction, left ventricular mass index, and systolic and diastolic blood pressure were higher in those who had received one of the older drugs. After adjustment for age only, GLS was significantly lower in those who were exposed to the more toxic ART drugs. Whilst there was a significant difference in diastolic function, as assessed by the ratio of early mitral inflow velocity and mitral annular early diastolic velocity (E/e'), and pulmonary pressures between those exposed to or not exposed to older mitochondrially toxic ART drugs, the values were still within the normal range for both groups. A large study of 662 men with HIV compared with 533 men without HIV from the Multicenter AIDS Cohort reported a lower LV mass in those currently receiving nucleoside reverse transcriptase (NRTI) containing ART, but they did not differentiate the older mitochondrial toxic ART and current exposure to less toxic NRTI (11).

The difference in heart rate between groups may be explained by a difference in fitness level. People without HIV had a higher LV mass index despite a lower blood pressure, which would, along with a lower heart rate, be compatible with increased cardiovascular fitness. There were minor differences in reported levels of exercise and more PWHIV reported exertional symptoms, which in the absence of differences in LV function and clinical evidence of cardiac failure or respiratory disease, might represent a reduced level of fitness. All PWHIV were under regular medical review, and none had physical findings thought to be due to heart failure. As in the general population, cardiorespiratory fitness has been observed to decline with age in PWHIV unrelated to the duration of HIV or immunological parameters (36).

Whilst previous echocardiographic studies have reported impairment in both systolic and diastolic function in PWHIV, there are a number of differences between the current study and previous reports. Several, but not all, studies contained no control group and referenced results against population data (6, 8, 9, 27, 28). It should be noted that GLS and measures of diastolic function in PWHIV in our study did not fall outside the population normal ranges. Our cohort of PWHIV comprised metropolitan dwelling Caucasians whereas some previous studies had more racially diverse populations (28). Similarly, several CMR studies reporting impaired LV function in people with HIV have not had comparable patient characteristics. Thus, those in Robbertse et al. were treatment naïve and early in their course of HIV, and the majority (80%) were of Black race. The study by Yan et al. included PWHIV of Asian (Chinese) race with an average age of 37 years. The largest study from the MACS cohort (all men, median age 58 years, 30% of Black race) revealed an independent association between HIV and greater LV mass index, LAV and RV size, lower RV function, but not LV ejection fraction (11). Although PWHIV in our study had been diagnosed with HIV a median of 14 years previously, and had a 12 year history of treatment with ART, they were taking contemporary, potent, and well-tolerated ART with excellent virological control and maintained good CD4 cell counts.

Diastolic left ventricular dysfunction has been reported to occur in PWHIV regardless of receipt of ART (37). The participants in our study were, by design, free of known cardiovascular disease, and the exclusion of those with a history of symptomatic cardiovascular disease may have diminished the magnitude of differences in cardiovascular function between people with and without HIV.

There is only one retrospective study that has assessed changes in cardiac structure and function longitudinally over 5 years, and these were performed in patients referred for echocardiography, so potentially a biased group. Whilst LV GLS, mass, and volumes were associated with markers of HIV disease control early in the course of infection (28), the authors established that virologically suppressed PWHIV had more normal GLS throughout, whilst LV mass index increased regardless of HIV viral load or level of immune dysfunction. There were no significant trends in other markers of cardiac function, suggesting that early virus suppression is associated with improved cardiac structure and function with ART (35).

### Limitations

There are a number of study limitations to consider. This was a small cross-sectional study of virologically suppressed, predominantly Caucasian men with HIV and no history of pre-existing cardiovascular disease. Thus, the findings may not be generalisable to the greater population of PWHIV. In addition, it is likely that people with significantly elevated pulmonary pressures may have been excluded by our study inclusion criteria. Whilst the groups were well matched for age and gender, there were significant differences in some other parameters that may have contributed to the findings. The study is strengthened by a single cardiologist performing and analysing the echocardiograms on all participants, in a single centre.

### Future directions

As the majority of studies have been cross-sectional, it is imperative to understand changes in cardiac structure and function over time. The impact of immediate initiation of ART at HIV diagnosis, level of immunodeficiency, and exposure to contemporary well-tolerated ART on parameters of ventricular function needs further characterisation but supports earlier diagnosis and treatment of HIV.

In summary, in this cohort of PWHIV with good virological control after 12 years of ART, we found no difference in left ventricular GLS compared with controls, which was within the reference levels for the general population. Despite the observed increased risk of clinical heart failure, and an increased prevalence of systolic and diastolic dysfunction reported in some echocardiographic and cardiac magnetic resonance imaging studies in PWHIV, our study does not support routine screening of asymptomatic individuals with echocardiography in people with HIV and similar characteristics to those presented in this study.

## Data Availability

The raw data supporting the conclusions of this article will be made available by the authors, without undue reservation.
